# Complete genome sequence of a carbapenem-resistant *Enterobacter hormaechei* strain harboring a novel conjugative IncHI2 plasmid carrying *bla*_IMP-6_ in Tokyo, Japan

**DOI:** 10.1128/mra.01451-25

**Published:** 2026-02-18

**Authors:** Kotaro Aoki, Kotaro Imaizumi, Yoko Mano, Kazuhiro Tateda, Nobuhiko Furuya

**Affiliations:** 1 Department of Microbiology and Infectious Diseases, Toho University School of Medicine, Tokyo, Japan; 2 Health Care Science, Graduate School of Bunkyo Gakuin University, Tokyo, Japan; Wellesley College, Wellesley, Massachusetts, USA

**Keywords:** carbapenemase-producing *Enterobacterales*, antimicrobial resistance, plasmids, IMP-6

## Abstract

We report the complete genome sequence of a carbapenem-resistant *Enterobacter hormaechei* ST133 strain isolated from a clinical stool sample at a hospital in Tokyo, Japan, in 2022. This isolate harbors a novel conjugative IncHI2-pST1 plasmid carrying *bla*_IMP-6_. Additionally, this plasmid co-carries *bla*_CTX-M-9_, *bla*_SHV-12_, *aac(6′)-IIc*, *sul1, aadA2*, *qnrA1*, *dfrA16*, and *mcr-9.2*.

## ANNOUNCEMENT

The spread of antimicrobial-resistant gram-negative rods (AMR-GNRs) is a global public health concern (1). Dissemination occurs through clonal expansion of successful lineages and horizontal transfer of AMR-carrying plasmids across species (2). Carbapenemase production is a primary mechanism of resistance, and the epidemiology of carbapenemase-producing *Enterobacterales* (CPE) varies by country and region (3). In Japan, IMP-type carbapenemases predominate among CPE isolates. Historically, IMP-1 emerged and spread first, followed by the emergence of IMP-6, which has now reached a prevalence comparable to that of IMP-1 (4). Plasmids carrying *bla*_IMP-1_ belong to various incompatibility (Inc) groups (e.g., IncHI2, IncFIB, IncW, IncN, and IncL/M), whereas *bla*_IMP-6_ is found exclusively on IncN plasmids (5–8). Here, we report the complete genome sequence of a carbapenemase-producing *Enterobacter hormaechei* sequence type (ST) 133 strain harboring a novel conjugative IncHI2 plasmid carrying *bla*_IMP-6_. The strain was isolated in 2022 from a routine stool sample at a hospital in Tokyo. The isolate grew on BTB agar (Kyokuto Pharmaceutical Industrial Co., Ltd., Japan) and demonstrated resistance to meropenem by the broth microdilution method (9, 10). This study was conducted in accordance with the principles of the Declaration of Helsinki.

The strain was recovered from a frozen stock stored at −80°C by culturing on Mueller-Hinton agar (MHA; Becton, Dickinson and Company, USA) at 35°C overnight. Genomic DNA was extracted for long-read sequencing using the MagLEAD system (Precision System Science Co., Ltd, Chiba, Japan). Libraries were prepared with the Rapid Barcoding Kit 24 V14 (SQK-RBK114.24) and sequenced on a MinION R10.4.1 flow cell (Oxford Nanopore Technologies, Oxford, UK). We used Dorado version 1.1.1 (model: dna_r10.4.1_8.2_400bps_sup@v5.2.0) for basecalling and demultiplexing. Low-quality reads (<Q15) and short reads (<5,000 bp) were filtered using Fastplong v0.4.1 (11). Assembly was conducted using Flye v2.9.6 (12) with reads error-corrected by DeChat v1.0.1 (13), followed by polishing with Medaka v1.11.3 (14). Genome annotation and bacterial species identification were conducted using DFAST v1.2.0 (15). Multilocus sequence type (MLST), antimicrobial resistance genes, plasmid Inc group, and plasmid MLST (pMLST) were identified using MLST v2.0 (16), ResFinder v4.7.2(17), PlasmidFinder v2.1 (18), and pMLST v2.0 (19), respectively. The novelty of the plasmid was assessed by Nucleotide BLAST against the Core nucleotide database (core_nt)(20). Conjugation assays were performed as previously described (5).

Whole-genome sequencing using the MinION platform yielded 1,394,922 reads with an *N*_50_ of 6,489 bp. The complete genome statistics and identified AMR genes of *E. hormaechei* TUM26385 are summarized in Table 1. Nucleotide BLAST analysis of pMTY26385_IncHI2 using default parameters returned no hits. pMTY26385_IncHI2 was more than 80 kbp smaller than the previously reported *bla*_IMP-1_-carrying IncHI2-pST1 plasmid, pMTY11043_IncHI2 (368,940 bp, GC 47.3%), identified in *E. hormaechei* ST78 in Tokyo in 2007 (5). Alignment analysis showed that pMTY26385_IncHI2 covered only 67.1% (247,403/368,940 bp) of the pMTY11043_IncHI2 sequence with ≥98% identity. pMTY26385_IncHI2 possessed a class 1 integron consisting of the 5′ conserved sequence (CS)-*bla*_IMP-6_-*aac(6′)-IIc-sul1*-3′CS. The structure of this integron differed from that of pMTY11041_IncHI2 only by the presence of *bla*_IMP-6_ instead of *bla*_IMP-1_. Our findings suggest that pMTY26385_IncHI2 was not directly derived from pMTY11043_IncHI2 and that pMTY26385_IncHI2 likely acquired the *bla*_IMP-6_-containing class 1 integron independently.

**TABLE 1:** Genomic characteristics and putative antimicrobial resistance genes of *Enterobacter hormaechei* TUM26385^*b*^

Features	Chromosome	Plasmid (pMTY26385_IncHI2)
GenBank accession number	JBSOSF010000001	JBSOSF010000002
Length (bp)	4,998,669	280,638
GC content (%)	55.4	46.4
Number of CDSs	4,716	309
AMR genes^*a*^	None	*bla*_IMP-6_*, bla*_CTX-M-9_*, bla*_SHV-12_*, aadA2b, aac(6′)-IIc, qnrA1, sul1, dfrA16,* and *mcr-9.2*

^*a*^ Although carbapenem resistance conferred by *bla*_IMP-6_ in this strain was confirmed by antimicrobial susceptibility testing, the other AMR genes remain putative because their corresponding phenotypes were not experimentally verified.

^*b*^ AMR, antimicrobial resistance; CDS, coding sequence.

## Data Availability

The sequence data for *E. hormaechei* TUM26385 have been deposited in GenBank under BioProject accession number PRJNA1236865, and the Sequence Read Archive under accession number SRR36718676.
